# A novel strategy to optimize critical information on over the counter labels for older adults

**DOI:** 10.1002/hsr2.1062

**Published:** 2023-01-25

**Authors:** Mark W. Becker, Deborah A. Kashy, Alyssa Harben, Krishnaa Venkatesan, Andrew Rodriguez, Matt Kebede, Beth Martin, Robert Breslow, Laura Bix

**Affiliations:** ^1^ Department of Psychology, Cognitive Neurology Group Michigan State University East Lansing Michigan USA; ^2^ School of Packaging Michigan State University East Lansing Michigan USA; ^3^ School of Pharmacy University of Wisconsin Madison Wisconsin USA

**Keywords:** improved adherence, medication error, patient safety, patient‐centered design

## Abstract

**Background and Aims:**

Labels designed to communicate critical information are paramount for the safe and effective use of over‐the‐counter medications; in recognition of this, the content and formatting of over the counter (OTC) labels sold in interstate commerce has been regulated for decades. Yet, available studies suggest that consumers frequently rely on limited information during decision making, failing to access the information required in the Drug Facts Label. This is particularly important for older consumers, who are at greater risk for adverse reactions to medicines. In two experiments we objectively evaluate how novel label designs that employ highlighting and a warning label placed on the package's front impact older consumers' attention to, and use of, critical information.

**Methods:**

In Experiment 1, 68 OTC patients (65+) engaged with a computer‐based task answering yes/no scenario‐based questions about a drug's appropriateness. In Experiment 2, 63 OTC patients (65+) conducted a forced‐choice task where one of two drugs presented on a computer screen was appropriate for a provided scenario while the other was not. Both tasks required participants to access and use critical label information (i.e., warnings or active ingredients) to respond correctly. Dependent variables analyzed were the proportion of correct responses and time to correct response.

**Results:**

Highlighting or placing critical information on the front of the package significantly improved response accuracy and time to correct response in Experiment 1 as compared to responses utilizing the standard label. For Experiment 2, participants were faster and more accurate when critical information was highlighted.

**Conclusions:**

Results provide direct measures of the efficacy of novel labeling strategies. This information is relevant for regulations which dictate label design in ways that enhance ease and safety of use of medications for older adults.

## 1 INTRODUCTION

Across the United States, the estimated, annual savings associated with over the counter (OTC) drug use is $146B ($52B saved in drugs costs and $95B in clinical savings).^1^ These savings, combined with an ever‐increasing range of readily available products used to treat a variety of conditions, have undoubtedly led to the recent growth experienced by the OTC market. Sales of OTC medicines more than doubled in the period between 2000 and 2020, from $14.7B in 2000 to $36.5B in 2020. OTC Monograph reform, which opens the door for more timely and flexible OTC regulation; the restoration of eligibility of OTCs under tax‐preferred HAS and FSA accounts; as well as increased trepidation about formal healthcare and financial concerns exacerbated by the pandemic have led experts to predict robust, continued growth for the OTC sector.^2^

As healthcare costs continue to rise and life spans increase,^3^, ^4^ those 65 and older are turning to OTCs as part of their self‐care regimens with increasing frequency. They represent the highest per capita users of OTC drugs,^5^ and it has been estimated that 25% of all older adults take a combination of 10 or more medications (both OTC and prescription) daily.^6^ This is concerning, as the likelihood of an Adverse Drug Reaction (ADR) is 10% when taking a single drug but jumps to 70%–75% for people who engage in polypharmacy,^7^ defined as taking five or more medications per day. ADRs are “noxious, unintended and undesired effect[s] of a drug, excluding therapeutic failures, intentional and accidental poisoning and drug abuse.”^8^ Aging consumers are at increased risk for ADRs for varied reasons, including changes in pharmacokinetics, pharmacodynamics, perception, cognition, and motor skills, increased prevalence of poor health literacy and inappropriate prescribing and monitoring practices specific to this population.^5^, ^9^, ^10^, ^11^, ^12^, ^13^, ^14^, ^15^ These issues are further compounded by a propensity for multimorbidity which catalyzes complex regimens that include a mix of prescriptions, OTCs, and supplements. Studies suggest that ADRs are the cause of 6%–12% of hospital admissions for older adults, serving as a major cause of morbidity and mortality for this population and that misuse of OTCs is very common for this population.

Being able to determine what OTC medication is appropriate for a unique individual's self‐care regimen represents a complex decision‐making process. Older consumers must take on the role of health care provider and make decisions about: symptoms to be treated, treatment options to consider, as well as how each option is situated within a complex mix of comorbid health conditions and existing treatments and dietary habits.^16^, ^17^

One strategy for helping consumers (of all types) make informed decisions related to OTC products is the use of a standardized “Drug Facts Label” (DFL) required on most OTC medicines sold in the US since 2002. The information content and formatting of the DFL are dictated by Title 21 of the Code of Federal Regulations (CFR)201.326.^18^ While labeling is important for all healthcare products, it's particularly important for OTC products as they lack the “learned intermediary” (i.e., prescribing physician and dispensing pharmacist) that is guaranteed to provide oversight for prescription products.^19^ The standardized DFL represents government efforts to provide consumers with critical information in an accessible format that is readily available at both the point of purchase and time of use.

That said, there are noted problems with the DFL which can be particularly problematic for older adults. Published difficulties with existing labels include the DFL's use of small font sizes^20^ with densely presented information,^20^ as well as the relative prioritization of non‐safety related information.^21^, ^22^ Failure to access information critical to safe product use (i.e., contained within the DFL) is a noted problem.^22^ A survey conducted by Aker et. al. suggests that consumers heavily rely on brand name, with very few considering current drug regimens or existing diagnoses during their decision‐making process when selecting an OTC.^23^ Eye tracking studies suggest that very few older adults even access the DFL when answering questions regarding an OTC's appropriateness for their use.^22^, ^23^, ^24^

In light of these findings, the overarching objective of this study is the development of label designs that positively impact older consumers' attention to, and use of, critical information compared to the existing, commercial standard.

We proposed to investigate the efficacy of a novel Front of Pack (FOP) labeling strategy for OTC products. FOPs are being employed increasingly throughout the globe on food packages. Although their formats vary, FOPs present truncated nutrition information, most commonly nutrients associated with disease states (e.g., fat, salt, sugar, calories), on the package's Principal Display Panel (PDP), the panel typically displayed at retail. A growing body of research suggests that FOPs garner attention readily and result in healthier food selections (see Hawley for a review).^25^ Labeling research also provides empirical evidence regarding the use of color for enhancing attention to critical information and speeding product differentiation.^26^, ^27^, ^28^

**Figure 1:**
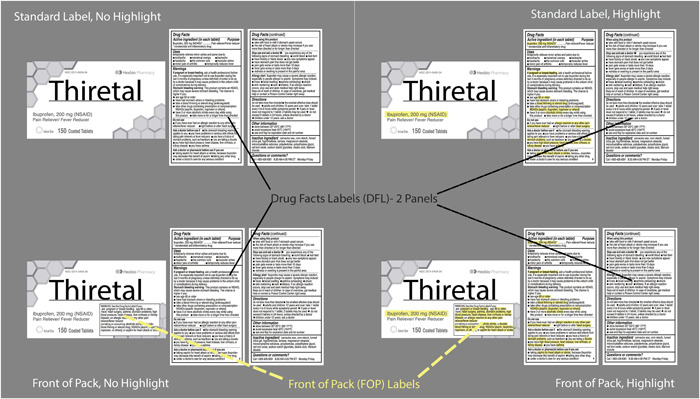
Four experimental treatments with basic terminology.

Leveraging what is known from the literature, we propose a more effective design for OTC labels, and objectively evaluate this approach. We test four OTC label formats (See Figure 1) which form a factorial combination of 2 (front of pack/standard label) × 2 (highlighting/no highlighting). Information prioritized as critical to the safe and effective use of these products was selected based on a survey of pharmacists conducted by Martin et al. Specifically, active ingredient (AI) and drug‐disease or drug‐drug contraindication warnings (DD) were moved to the FOP and highlighted in the relevant treatments.^29^

## 2 RESEARCH DESIGN AND METHODS

Two experiments objectively assessed our novel designs against the standard, commercial practice. Experiment 1 utilized an Absolute Judgment Task and Experiment 2 employed a Forced‐Choice Task.

Methods were approved by the Michigan State University (MSU) Psychology and Social Science Internal Review Board under application IRB STUDY00000832. Participants in Experiment 1 were recruited utilizing programming offered by MSU Extension which focused on older adults from multiple counties in the state using fliers that were distributed during existing programming targeting the population of interest. Participants for Experiment 2 were recruited through RSVP of Ingham, Eaton and Clinton counties, part of the AmeriCorps seniors volunteer program.

Participants who were interested contacted the research team via a phone number on the flier to verify eligibility and schedule the research. To be eligible to participate they had to: manage their own medication, be aged 65+, be legally sighted, and have consumed at least 1 OTC medication in the past year. Once eligibility and interest was confirmed, researchers scheduled a convenient time and provided directions to the test location. Follow up, reminder calls were made 24 h before the appointment.

Upon arrival at the research location, an informed, written consent process was utilized for the collection of all data which identified participants only by subject number. During the consent process, the research team provided a brief explanation of the activities comprising the experiment, including the benefits, risks, and time commitment. All participants were told that they could discontinue at any time or opt out of any portion of the research and still receive the $50 incentive. Participants were then provided the approved consent form and provided time to enable their review and signature of the document.

### 2.1 Participant characterization

Participants were characterized using: a Short Blessed Test^30^; basic demographic information; a Rapid Estimate of Adult Literacy in Medicine ‐ Revised (REALM‐R)^31^; a near point visual acuity test (Sloan Pocket Size Near Vision Card with Continuous Text by Precision Vision in Woodstock IL) and the ability to see color (Pseudo‐Isochromatic Plates by Richmond Products, Southeast Albuquerque NM). We also collected self‐reported familiarity of common OTC active ingredients and brands.

### 2.2 Power calculations

Power estimates were based on our prior work investigating medical device labeling in which participants made a speeded selection between two packages given a criterion (i.e., was latex free).^32^ Across trials, critical information (latex, expiration date, sterility, etc.) was highlighted or not. The highlighting effect size was *d* = 0.84. However, that sample was comprised of younger experts (i.e., medical professionals). Anticipating that our older participants would be more variable, we based our power calculation on *d* = 0.42 (50% of the observed d). A sample of 60 participants (75 recruited with 20% attrition) for each experiment was predicted to allow us to detect *d* = 0.42 with power >0.85.

### 2.3 Stimulus and experimental set up

Experiment 1 consisted of a series of absolute judgment trials that included a single OTC label presented in one of the four treatment formats (see Figure 1) with a scenario‐based question that required information from the label to be answered correctly in binary fashion (Yes/No) as quickly as possible. Experiment 2 asked which of two products presented on a computer screen was appropriate for use based on a posed scenario. Each of the two OTC labels appearing in the same trial were presented with the same treatment level (see Figure 1) in addition to a question (e.g., which product is appropriate for someone who has been diagnosed with hypertension?). Response variables were the proportion of correct responses and the time to correct response for both experiments.

### 2.4 Statistical methods

Analyses were conducted separately for each of two types of critical information (AI and DD). This was done because of the imbalance in the prominence of AI information. AI already appears on the front of the package, generally in a much larger font than what appears in the DFL (see Figure 1); as such, it is inherently different from the other critical information, warnings we have termed DD. When we created FOP label treatments (which incorporated DD information), we size matched the information to the warning information that already appeared in the DFL.

For Experiment 1, we also analyzed the trials where “yes” was the correct response separately from the trials where “no” was the correct response. Trials where yes was the correct response comprised cases where the warning label suggested that a medication should not be used. For instance, for the prompt, “Should someone avoid this product right before or after heart surgery”?, a yes/correct trial would consist of a label with an explicit warning statement relating to the danger of using the product near the time of a surgery. By contrast, the “no‐correct” trials were trials where the product was not contraindicated for use by warnings. For instance, the same question prompt about avoiding the medication right before or after heart surgery would appear with a drug that is not contraindicated with such procedures. However, because of the way warnings work, a drug that is safe to take near a heart surgery does not explicitly state that the drug is safe in that circumstance. Instead, the label would not include related surgery information at all, changing the task into a search for the absence of information.

These two cases are analogous to target‐present and target‐absent trials during a typical visual search task. Within the visual search literature, these types of trials are analyzed separately because they are different tasks. When a target is present, search activities only need occur until the point the relevant information is found. However, to determine that a target is not present anywhere in the display, participants need to search the entire display and be certain that they have not missed the information. Thus, typical reaction times are usually far quicker for target‐present than target‐absent responses. “Target‐present trials” provide an indication of how quickly and accurately people access critical warnings when present; thus, comparing participant responses related to accuracy and time across label designs represents an objective way to evaluate the efficacy of differing approaches to warning design. This was not the case for Experiment two, where one of the pair of OTCs presented in each trial was always contraindicated.

For both experiments, we calculated the median reaction time (RT) and percent correct for each subject for each condition. Linear Mixed Models with the fixed effects of warning label type (standard/FOP warning) and highlighting (highlighted/not highlighted) and the covariates of: sex, education level (as a binary variable of at least some college, or high school or less), race (as a binary variable of white, non‐Hispanic or an individual from another racial or ethnic group), age, Near Point Visual Acuity, and Health Literacy (in the form of participants' Realm R score) were used for both the reaction time and percentage correct accuracy analyses. Follow‐up simple effects were Bonferroni corrected for multiple comparisons. Repeated measurements for participants were modeled as compound symmetry.

## 3 RESULTS/FINDINGS

### 3.1 Experiment 1—absolute judgment task—sample description

Seventy‐five older adults participated in Experiment 1; 2 were dismissed due to ineligibility associated with screening criteria (under age 65), 4 were dismissed due to scores on the Short‐Blessed Test which indicated an inability to provide informed consent, and 1 withdrew from the experiment early due to technical difficulties. The analyzed sample comprised of responses from 18 men and 50 women who ranged from 65 to 88 years old (*M* = 71.8, standard deviation [SD] = 5.9). The racial and ethnic background of the sample was 58.8% White, 35.3% Black, 1.5% Asian, and 1.5% Native American, with 2.9% of participants reporting being Hispanic or Latino. One participant (1.5%) had no high‐school, 31 (46%) had some high‐school, 15 (22%) had some level of Associates, 11 Bachelors experience (16%), 7 Masters experience (10%) and 3 some level of Doctoral education (4%). Their reported median annual household income was $21,000 ranging from $1,300–250,000.

### 3.2 Experiment 2—forced choice task—sample description

Of the 66 participants recruited, 2 were dismissed due to scores on the Short‐Blessed Test, and one failed to finish due to a technical issue. Of the final sample, 44 participants were female and 19 were male, of which 14 had completed some level of High School (22%), 13 some level of Associates (21%), 16 a Bachelors (25%), 12 a Masters (19%) and 8 some level of Doctoral education (13%); all but three reported their primary language as English. The median annual income was $52,000, ranging from $35,510–160,000. The vast majority self‐reported as white (*N* = 59; 94%) two participants self‐identified as black or mixed race (3%), two as Asian (3%). For participants who reported their age (*n* = 65), they ranged from 65 to 88 years (average=74.2, SD = 5.3).

### 3.3 Experiment 1—absolute judgment task results

#### 3.3.1 Finding critical warnings in contraindicated OTCs

**Figure 2:**
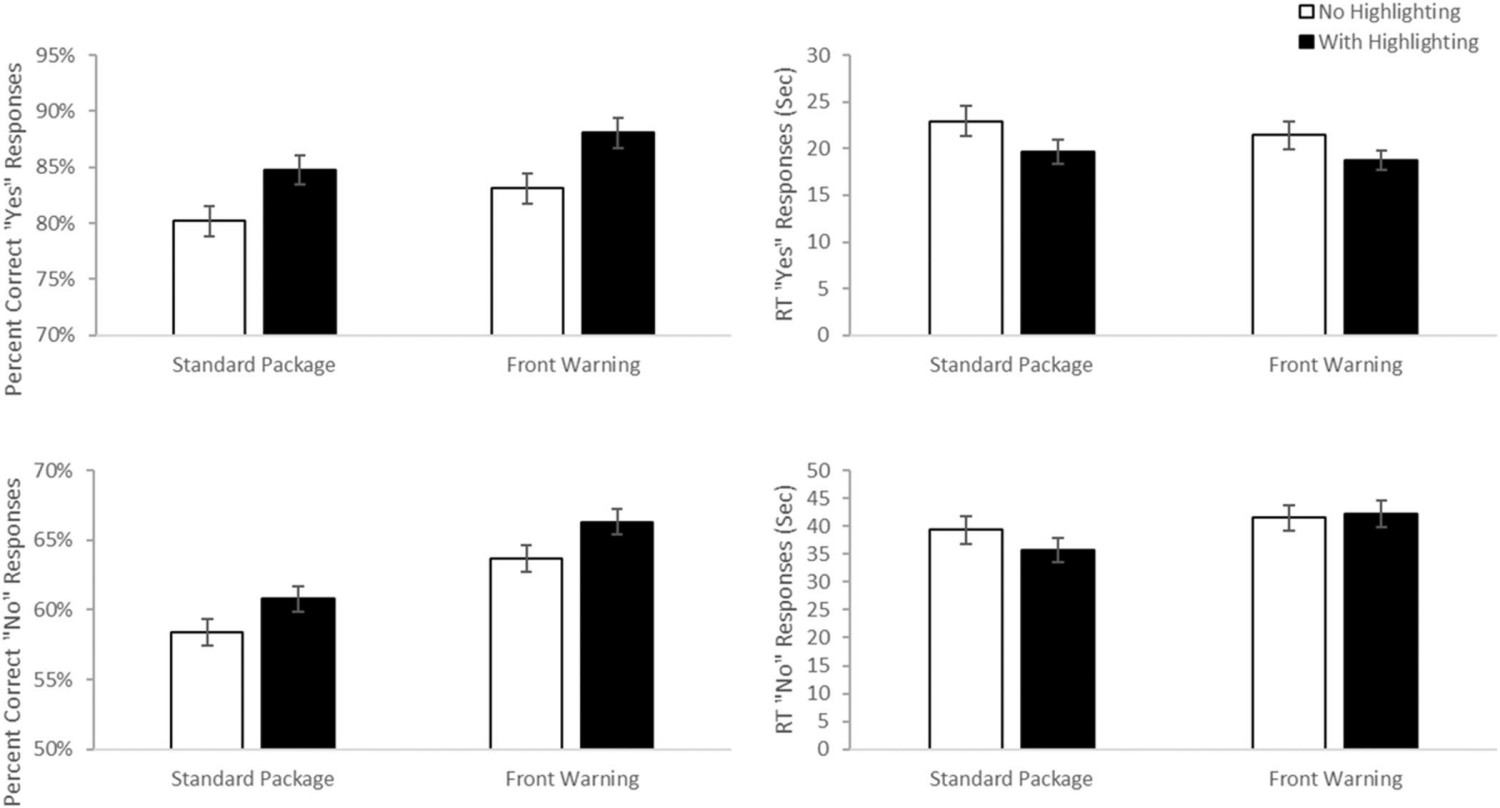
Experiment 1—absolute judgment warning results. Mean accuracy (left panels) and reaction times to make correct responses (right panels) are presented for trials where the medication was contraindicated (a “yes” response was correct) top panels) and where the medication was not contraindicated (a “no” response was correct lower panels). Error bars are the standard error of the mean.

Arguably, trials where medication use is contraindicated (see Figure 2 upper panels), the “yes” correct trials, are the most critical. These trials allow us to determine how our label manipulations influence people's ability to successfully identify OTCs that should be avoided. When analyzing accuracy, we found a main effect of label type, F(1, 198) = 5.56, *p* = 0.019, *d* = 0.34, with better performance with a front warning label than a standard label (M difference = 2.8%, 95% confidence interval [CI] 0.46 – 5.2%). There was also a main effect of highlighting, F(1, 198) = 17.850, *p* < 0.001, *d* = 0.60, with better accuracy with highlighting than without (M difference = 5.1%, 95% CI 2.7%–7.5%). There was no evidence of a significant interaction, F(1, 198) = 0.071, *p* = 0.79, *d* = 0.04, suggesting that both factors independently influence response accuracy. A similar analysis on median Reaction Time for correct trials found a main effect of highlighting, F(1, 191.7) = 14.566, *p* < 0.001, *d* = 0.55, with significantly faster response times for highlighted trials (M difference = 3.01 s, 95% CI 1.46–4.57 s). While there was a numerical trend for faster RTs with the front warning label, the main effect of label type did not reach significance, F(1, 192.17) = 1.54, *p* = 0.22, *d* = 0.18. Again, no evidence of significant interaction between the two factors, F(1, 191.7) = 0.644, *p* = 0.423, *d* = 0.12, was identified. In short, these are fairly ideal data. Both highlighting and the use of a front of pack warning increased accuracy and, at least for highlighting, decreased the time participants took to key a correct response.

#### 3.3.2 When warnings do not contraindicate

As mentioned above, when a medication was not contraindicated with the scenario (a “no” response), participants searched for information in a “target absent” environment (See Figure 2—bottom panels). There was a main effect of label type, F(1, 198) = 16.22, *p* < 0.001, *d* = 0.57 on response accuracy, with better accuracy associated with trials involving a front of pack warning label (mean difference = 5.33%, 95% CI 2.72%–7.94%). A main effect of highlighting was also identified, F(1, 198) = 4.17, *p* = 0.04, *d* = 0.29, with better accuracy in trials containing highlighting than those without (mean difference = 2.70%, 95% CI 0.09%–5.3%). No evidence of a significant interaction, F(1, 198) = 0.002, *p* = 0.96 was identified. A significant main effect of label type, F(1, 169.2) = 8.76, *p* = 0.004, *d* = 0.46, was identified related to reaction time, with slower responses when there was a front of pack warning label (mean difference = 5.22 s, 95% CI 1.74–8.70 s). There was no evidence of an effect of highlighting, F(1, 169.1) = 0.18, *p* = 0.67. *d* = 0.07 or an interaction, F(1, 169.1) = 1.33, *p* = 0.25, *d* = 0.18.

There are two things worth noting from these “no” trials. First, the fact that accuracy increases in the presence of a front warning label rules out a possible interpretation that the presence of a front warning label simply makes people more conservative and, thus, more likely to say that a medication should be avoided. If that were the case, then, in cases where the correct answer was the medication was acceptable would have decreased rather than increased. Second, it is worth noting that the overall accuracy is far lower and overall RT are far longer in these trials than in the “yes” correct trials. This is what one expects, given that these are searches for the absence of information and further justifies treating these trial types separately.

#### 3.3.3 Prompts requiring active ingredient information

Half of our prompts required utilization of the active ingredient (AI) rather than the drug/drug or drug/disease warnings (DD). As with the warning prompts, AI relevant prompts had both “Yes” correct and “No” correct trials, which, again, represented target present and target absent information, respectively. For instance, for the prompt “Does this contain Acetaminophen?” the “yes” correct trials involved a drug with acetaminophen displayed on the front panel and in the DFL. In the “no” correct trials involving this prompt, the drug would list a different active ingredient (e.g., Omeprazole) and the word acetaminophen would not appear anywhere on the labeling.

**Figure 3:**
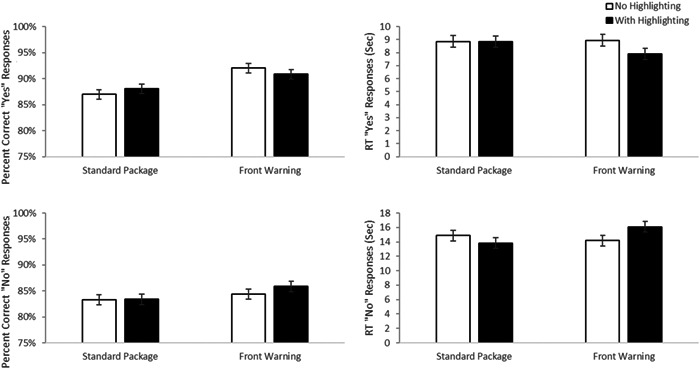
Experiment 1—absolute judgment active ingredient results. Mean accuracy (left panels) and reaction times to make correct responses (right panels) are presented for trials where the medication was contraindicated (top panels) and where the medication was not contraindicated (lower panels). Error bars are the standard error of the mean.

In the AI trials, the manipulations had little effect on performance (See Figure 3). For the “yes” correct trials accuracy, there was a main effect of label type, F(1, 198)  = 38.01, *p* < 0.001, *d* = 0.88, with better performance with the front warning label than without (mean difference = 5.32%, 95% CI 3.62%–7.02%). There was no evidence of a significant effect of highlighting, F(1, 198) = 0.421, *p* = 0.52, *d* = 0.09, nor an interaction, F(1, 198) = 1.06, *p* = 0.31, *d* = 0.15. For the dependent variable, reaction time, a significant main effect of label type was identified F(1, 196) = 4.13, *p* = 0.04, *d* = 0.29, with faster RTs in the presence of a front of pack warning (mean difference = 1.45 s, 95% CI = 0.04–2.85  s). There was no evidence for a main effect of highlighting, F(1, 195.1) = 1.73, *p* = 0.19, *d* = 0.19 or interaction, F(1, 195) = 0.036, *p* = 0.85, *d* = 0.03.

For the “no” correct trials, none of the main effects approached significance, all Fs < 2.4, all *p* > 0.12, all d < 0.21, for either accuracy or reaction time. Similarly, the interactions did not rise to the level of significance, both F < 3.11, both *p* > 0.08, both *d* < 0.26.

### 3.4 Discussion

When a warning suggests that an over‐the‐counter medication is contraindicated for a given scenario (the “yes” correct trials), both highlighting and the front of pack warning increased accuracy in using that information. Additionally, it did so while reducing the time to reach those correct decisions, suggesting that the improvement was not due to a speed accuracy tradeoff but, instead, that the use of these label manipulations makes accessing this critical information easier. In short, the data from these warning cases are ideal, providing a strong argument for the benefits of this novel approach.

The data from the trials which required accessing AI information are less compelling. However, it is worth noting that, overall, the ability to use AI information is superior to warning information, even with the standard label. Considering that the condition with the worst performance was the “no” response trials with the most difficult case being a standard label, participants were 83% accurate and took less than 15 s for prompts requiring AI information. By contrast, for that same difficult condition when warning information was required people were only 58% accurate and it took them almost 40 s, suggesting that standards designs need to improve access to, and use of, warning information. The fact that the label manipulations improve warning performance (DD) without costing a performance in the use of AI information argues that the approach should still provide an overall benefit.

### 3.5 Experiment 2—forced choice task—results

#### 3.5.1 Using warning information during forced‐choice selection

**Figure 4:**
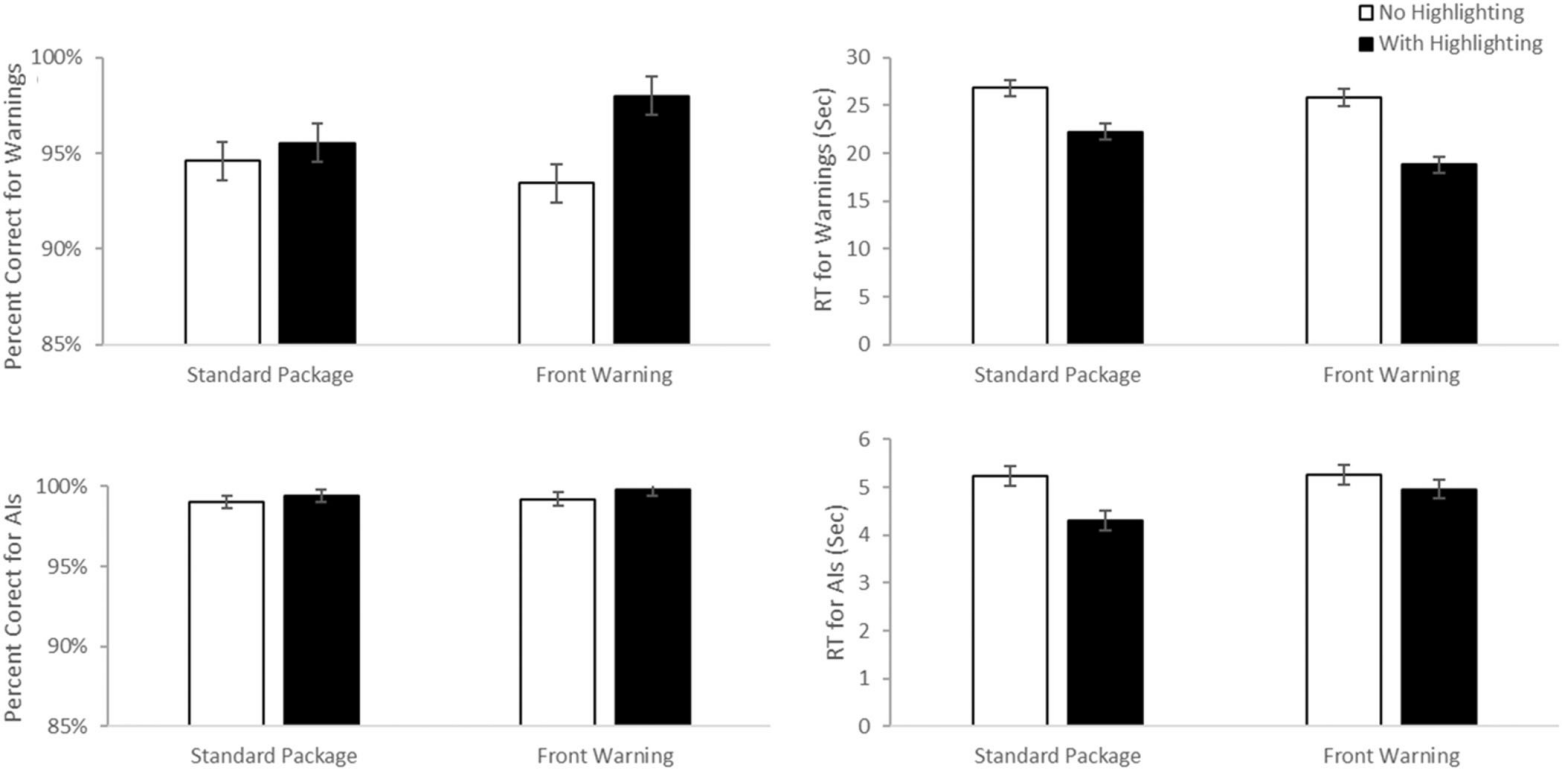
Experiment 2—forced choice selection results. Mean accuracy (left panels) and reaction times to make correct responses (right panels) are presented for trials where the critical infomration was Warning information (top panels) and where the critical informaiton was Active Ingredient information (lower panels). Error bars are the standard error of the mean.

Similar analyses were conducted using median reaction times and accuracy for the conditions where the prompt required the use of warning information to identify the correct product for a given scenario from a presented pair (See Figure 4—top panels). For accuracy, there was a main effect of highlighting, F(1, 183) = 7.73, *p* = 0.006, *d* = 0.41, with greater accuracy in the highlighted than unhighlighted conditions (M difference = 2.9%, 95% CI 0.84%–4.92%). There was no evidence of a significant main effect of label type, F(1, 183) = 0.306 *p* = 0.58, *d* = 0.08. There was a marginally significant interaction, F(1, 183) = 3.52, *p* = 0.06, *d* = 0.28. Paired comparisons reveal that the highlighted label with a front of pack warning performed better than the front warning label (FOP) label without highlighting, *p* = 0.007 (M difference = 4.8%, 95% CI 0.9%–8.7%). No other simple effects reached significance, all *p* > 0.11. It is worth noting that the overall accuracy was very high so the weakness of this effect in accuracy may have been impacted by ceiling effects.

Consistent with this argument, the analyses of median RTs found a main effect of highlighting, F(1, 183) = 36.70, *p* < 0.001, *d* = 0.90, with faster RTs for highlighted than unhighlighted displays (M difference = 5.68 s, 95% CI 3.83–7.54 s) There was also a significant main effect of label type, F(1, 183) = 5.33, *p* = 0.02, *d* = 0.34, with faster RT's for packages with a front warning (FOP) label (M difference  = 2.17 s, 95% CI 0.31–4.02 s) and no evidence that the two factors interacted, F(1, 183) = 2.36, *p* = 0.13, *d* = 0.23.

In short, these data replicate the findings from Experiment 1 that suggest that both factors add an independent benefit to people's ability to find and effectively use warning information.

#### 3.5.2 Using AI information during forced‐choice selection

We ran the same type of analyses on the accuracy and median RT data for the cases where identifying the appropriate medication depended on access to active ingredient information (See Figure 4—lower panels). For accuracy, overall performance was likely near ceiling for trials requiring AI information, and neither main effects or the interaction approached significance, all F(1, 183) <1.64, all *p* > 0.2, all *d* < 0.19. For Reaction time, there was a main effect of highlighting, F(1, 183) = 14.82, *p* < 0.001, *d* = 0.57, with faster RTS with highlighting than without (mean difference = 0.622 s, 95% CI 0.303–0.941 s). There was also a marginally significant main effect of label type, F(1, 183) = 3.78, *p* = 0.05, *d* = 0.29, however here the RTs were *slower* in the presence of an FOP. There was also a marginally significant interaction, F(1, 183) = 3.77, *p* = 0.05, *d* = 0.29. Follow‐up paired comparisons revealed that highlighted, standard labels produced faster RTs than the other three conditions, all *p* < 0.04 (all mean differences > 0.62 s, 95% CIs 0.018–1.55 s). None of the other three conditions differed from one another, all *p* > 0.8. Our interpretation of this pattern is that highlighting AI information speeds people's ability to find and use that information when it is important. However, the presence of a front label competes with the AI information for attention, and thus slows the ability of people to access and use AI information. The positive benefit of highlighting is canceled out by the competition for attention in the label that has both highlighting and a front of pack label.

## 4 DISCUSSION AND IMPLICATIONS

OTC packaging serves a vital role in facilitating safe and effective use of medication by older adults, a population identified as at risk for ADRs.

In response to this concern, the Consumer Healthcare Products Association (CHPA) and the Gerontological Society of America (GSA) assembled a panel of experts with the goal of identifying critical gaps in the “relatively neglected area of OTC use among older adults to promote safe and effective use of the same.”^33^ Among the key research priorities identified in the 2014 expert report was the development of “optimized and standardized labeling of OTC medications so information is presented in a format that is easily accessible to the aging population.”^34^ The call for this type of work is not limited to the GSA/CHPA efforts; a systematic review of studies investigating OTC labeling suggests the need for research ensuring consumers can “effectively find and understand information to facilitate safe and effective self‐management.”^34^

In the recent past, the FDA required either highlighting or increased font size of the active ingredient to improve communication of the risks related to specific drugs (acetaminophen and NSAIDs).^35^ Our results support the expansion of highlighting information beyond AI in a broader array of products. Research conducted by Goyal et al. suggested that highlighting increased risk perception related to products containing acetaminophen.^36^ In addition to increased risk perception of OTC products, our work suggests that highlighting critical information has the potential to enhance the usability of the DFL.

The studies also support that placing critical information on the package's front panel facilitates the use of not only warning information (DD), but also the active ingredients (AI). This is indicated by the finding that the presence of the critical warnings on front improved participants' accuracy in AI information trials in Experiment 1. The mechanism of how the presence of the critical warnings on front influenced information search is unknown and should be further explored with an experiment utilizing an eye‐tracking methodology to examine scan paths.

One suggestion for future work is replicating the methodology from the cross‐product comparisons utilized in Experiment 2 within a product category to better simulate the decision making of consumers in a retail environment. Requiring consumers to choose between two analgesics, two antihistamines, or two antacids would better replicate the types of decisions consumers of OTCs make regularly. As there are some active ingredients in product categories that are safer for older adults than others,^37^ this would also provide valuable insight into how to better communicate risks that increase with age: such as the risk of stomach bleeding, or the risk of an anticholinergic effect.

In conclusion, the work presented herein supports further investigation aimed at improving OTC medication labeling. Both our individual assessment and cross‐product comparison of OTC labels have provided evidence supporting the need for more ecologically valid research on consumer's use of optimized OTC labels, with the goal of improving the safety of OTC medication use.

## 5 LIMITATIONS

While the results of the studies presented in this article are promising, there are also inherent limitations to this work. The first limitation is the mock branding used in both studies. Because no real brands were used and potential color effects were controlled for using gray scale images, generalizability to the broader OTC market is limited. As branding is known to influence how consumers perceive medications,^38^, ^39^ these methods should be replicated with real brands to examine whether or not the results hold. Additionally, the mock brands that were utilized were grayscale, which could have increased the relative visual salience of the highlighting, biasing the results.^40^ Repeating these studies with branded stimuli would provide insight into whether or not the benefits of highlighting remain when the highlighting is not the only non‐grayscale component of the stimuli.

## AUTHOR CONTRIBUTIONS

**Mark W. Becker**: Conceptualization; data curation; formal analysis; funding acquisition; methodology; project administration; resources; supervision; visualization; writing – original draft; writing – review and editing. **Deborah A. Kashy**: Data curation; formal analysis; methodology; visualization; writing – review and editing. **Alyssa Harben**: Conceptualization; data curation; formal analysis; investigation; methodology; project administration; software; writing – original draft; writing – review and editing. **Krishnaa Venkatesan**: Investigation; methodology; project administration; software; writing – review and editing. **Andrew Rodriguez**: Investigation; methodology; project administration; software; writing – review and editing. **Matt Kebede**: Investigation; methodology; software; writing – review and editing. **Beth Martin**: Conceptualization; funding acquisition; writing – review and editing. **Robert Breslow**: Conceptualization; funding acquisition; writing – review and editing. **Laura Bix**: Conceptualization; funding acquisition; investigation; methodology; project administration; writing – original draft; writing – review and editing. All authors have read and approved the final version of the manuscript, Laura Bix (corresponding author) and Mark Becker (Manuscript Guarantor) had full access to all of the data in this study and take complete responsibility for the integrity of the data and accuracy of the data analysis.

## CONFLICTS OF INTEREST STATEMENT

Laura Bix has served as a consultant to Vertex Pharmaceuticals, Inc., a biotech firm focused on drugs to treat cystic fibrosis, providing expertise on the development of protocols to evaluate packaging ease of use. Laura Bix has received an honorarium and travel reimbursement to speak at Baxter and Johnson & Johnson Companies and travel reimbursement to share work from her group at the International Quality and Productivity Center's conference on Pharmaceutical Labeling. Travel reimbursement has also been provided by the US Food and Drug Administration and the US Centers for Disease Control and Prevention in support of her participation for ongoing efforts medication safety. Laura Bix and Mark Becker have served as CoPIs on work supported by the Consumer Healthcare Products Association; this funding did not support work presented herein. None of these entities played any role in the design or conduct of the study; collection, management, analysis or interpretation of the date; preparation, review or approval of the manuscript or the decision to submit or publish the study.

## ETHICS STATEMENT

All of the listed authors have contributed in meaningful ways (ICMJE guidelines) to the submission, have reviewed the manuscript and agreed to the order of authorship as published. The corresponding author is Laura Bix; she agrees to take responsibility for communications related to the submitted manuscript. The manuscript has not been published (in whole or part) elsewhere and is not under consideration elsewhere. The manuscript guarantor and lead author, Mark W. Becker, affirms that this manuscript is an honest, accurate and transparent account of the study being reported; that no important aspects of the study have been omitted, and that any discrepancies from the study as planned (and if relevant, registered) have been explained. Methods were approved by the Michigan State University (MSU) Psychology and Social Science Internal Review Board under application IRB STUDY00000832. An informed, written consent process was utilized for the collection of all data which identified participants only by subject number. Eligibility included: the ability to manage their own medication, aged 65+, be legally sighted, and have consumed at least 1 OTC medication in the past year. Participants in Experiment 1 were recruited utilizing programming offered by MSU Extension which focused on older adults from multiple counties in the state.

## TRANSPARENCY STATEMENT

The lead author Laura Bix affirms that this manuscript is an honest, accurate, and transparent account of the study being reported; that no important aspects of the study have been omitted; and that any discrepancies from the study as planned (and, if relevant, registered) have been explained.

## Data Availability

The authors agree to make the aggregated de‐identified flat file available upon request.
